# Early-stage measurable residual disease dynamics and IGHV repertoire reconstitution during venetoclax and obinutuzumab treatment in chronic lymphocytic leukemia

**DOI:** 10.1038/s41408-023-00870-2

**Published:** 2023-07-04

**Authors:** P. J. Hengeveld, J. Schilperoord-Vermeulen, M. Y. van der Klift, J. M. N. Dubois, P. M. Kolijn, F. G. Kavelaars, M. Rijken, J. A. Dobber, K. Nasserinejad, S. Kersting, P. E. Westerweel, A. P. Kater, A. W. Langerak, M-D. Levin

**Affiliations:** 1 Department of Internal Medicine, Albert Schweitzer Hospital, Dordrecht, the Netherlands; 2 Department of Immunology, Erasmus MC, Rotterdam, the Netherlands; 3 Department of Hematology and Experimental Immunology, Cancer Center Amsterdam, Amsterdam University Medical Centers, University of Amsterdam, Amsterdam, the Netherlands; 4 Department of Hematology, Erasmus MC Cancer Institute, University Medical Center Rotterdam, Rotterdam, the Netherlands; 5 HOVON Data Center, Department of Hematology, Erasmus MC Cancer Institute, Rotterdam, the Netherlands; 6 Department of Hematology, Haga Hospital, The Hague, The Netherlands

**Keywords:** Chronic lymphocytic leukaemia, Chronic lymphocytic leukaemia

Dear Editor,

Measurable residual disease (MRD) is an important post-treatment biomarker that predicts progression-free survival (PFS) following fixed-duration treatment in chronic lymphocytic leukemia (CLL) [1, 2]. MRD is often reported as a static parameter, cross-sectionally assessed at the end of treatment. However, as treatment is initiated and subsequently withheld, MRD levels follow an L-shaped trajectory, characterized by a rapid decrease followed by a delayed logistic regrowth phase. Successive MRD measurements, which more comprehensively capture MRD dynamics, may reveal additional prognostic information. Indeed, serial MRD measurements in the CLL14 and MURANO trials have demonstrated that compared to chemoimmunotherapy, treatment with venetoclax and anti-CD20 monoclonal antibodies reduced the rate of clonal regrowth [3, 4]. However, whether in the context of venetoclax and obinutuzumab, very early MRD measurements, performed shortly after treatment initiation, may already predict MRD levels and prognosis at later time points, has not been thoroughly investigated.

In this study, we used the IGHV leader-based next-generation sequencing (NGS) assay to longitudinally measure MRD in samples from the HOVON-139/GIVE trial [5, 6]. The HOVON-139/GIVE trial is a first-line randomized phase-II trial, intended to evaluate the efficacy and safety of pre-induction with two cycles of obinutuzumab, followed by induction with six cycles of obinutuzumab and venetoclax and six cycles of venetoclax monotherapy, followed by randomization to either 12 cycles of venetoclax maintenance or MRD-conditional treatment cessation [5].

Of the patients treated in the HOVON-139/GIVE trial, 60/67 had ≥1 sample available for MRD measurement (Supplementary Table 1). In this cohort, 28 patients were randomized to receive an additional year of venetoclax consolidation, 27 were randomized to receive MRD-conditional consolidation treatment, four patients went off protocol at the time of randomization and one patient died before randomization (Supplementary Fig. 1). In the consolidation arm, 28/28 patients received the additional 12 cycles of venetoclax. In the MRD-conditional arm, 26/27 did not receive additional venetoclax consolidation, whereas one patient received 8/12 cycles of venetoclax consolidation. To allow a per-protocol comparison of a venetoclax consolidation arm to a no consolidation arm, we censored this patient at the time of randomization. The four patients who went off protocol at the time of randomization were included in the no consolidation arm, as they had not received any further anti-leukemic treatment. The median follow-up was 43 months (IQR 38–50).

MRD was measured in 185 samples, representing four time points: during the fourth week of venetoclax ramp-up in induction cycle 1, at the end of the twelfth cycle of induction treatment (EOiT), and 6 and 12 months post-randomization (R+6 and R+12) (Supplementary Fig. 1). In 131/185 (71%) samples, MRD was undetectable (<10^−5^). MRD dynamics for the total cohort are depicted in Fig. 1A. MRD was undetectable (<10^−5^) in 7/36 (19.5%) patients in induction cycle 1 (C1), 40/43 patients (93.0%) at EOiT, 45/53 (84.9%) patients at R+6 and 39/53 (73.6%) at R+12. MRD levels were comparable between the randomization arms and are depicted in Supplementary Fig. 2A,B. An analysis of the interassay agreement between the IGHV leader-based NGS assay and a multicolor flow cytometry approach is available in the Supplementary Data.

**Fig. 1:**
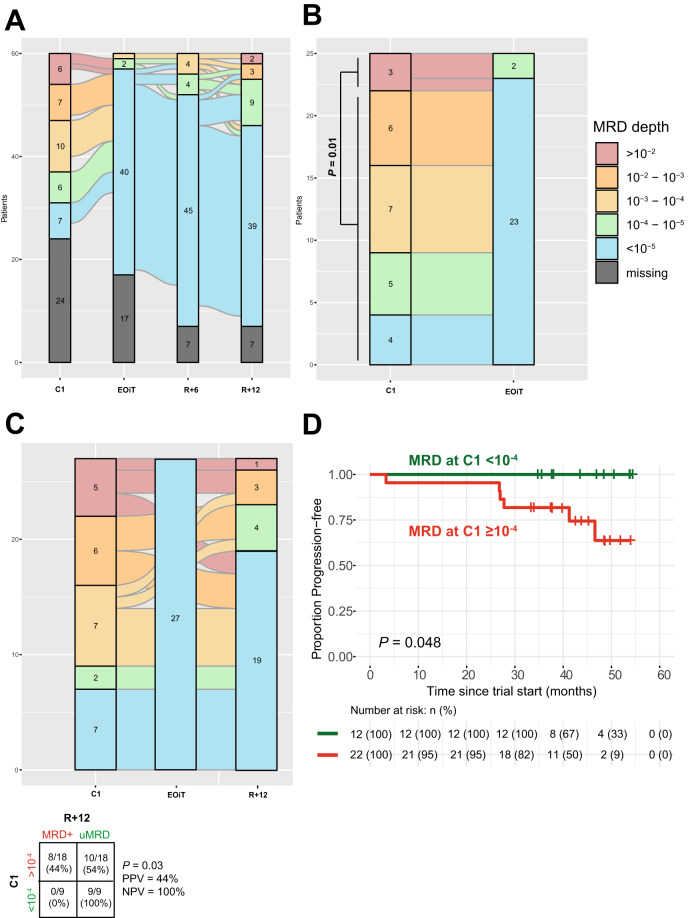
MRD dynamics in the HOVON-139/GIVE trial. Alluvial plots depicting MRD dynamics in the complete cohort of the HOVON-139/GIVE trial (*n* = 60) (**A**), or depicting MRD dynamics between venetoclax ramp-up and either the end of induction treatment (**B**) or 1 year after randomization (**C**). Counts indicate absolute numbers; ribbon sizes represent proportions. Statistical significance was evaluated using Fisher’s exact tests. **D** Kaplan–Meier plot and risk-table estimating progression-free survival in the HOVON-139/GIVE trial, stratified by MRD depth above or below MRD 10^−4^ during venetoclax ramp-up. Crosses indicate right-censoring. Statistical significance was evaluated using a log-rank test. C1 induction cycle 1, EOiT end of induction treatment, MRD measurable residual disease, MRD+ detectable measurable residual disease, R+6 6 months after randomization, R+12 12 months after randomization, NPV negative predictive value, PPV positive predictive value, uMRD undetectable measurable residual disease.

In the fourth week of induction cycle 1, following 11 weeks of treatment, MRD levels ranged from an upper bound of 3.5*10^−1^ (35% of PBMCs) down to MRD < 10^−5^ in 7/36 (19%) patients. In patients with MRD measurements available at both C1 and EOiT (*n* = 25), high levels of MRD (>10^−2^) at C1 were associated with failure of reaching MRD < 10^−5^ at EOiT (2/3 versus 0/22, *P* = 0.01) (Fig. 1B). In addition, in patients who achieved MRD < 10^−5^ at EOiT and with MRD measurements available at C1 and R+12 (*n* = 27), loss of MRD < 10^−5^ at R+12 exclusively occurred in patients with MRD ≥ 10^−4^ at C1 (8/18 vs 0/9, *P* = 0.03, PPV 44%, NPV 100%) (Fig. 1C), suggestive of a shallow depth of response in this group. There was a positive rank correlation between the MRD level at C1 and at R+12 (Spearman’s *ρ* = 0.48, *P* = 0.01). Most importantly, no patient with MRD < 10^−4^ at C1 experienced disease progression during follow-up, resulting in superior PFS at 40 months, compared to patients with MRD ≥ 10^−4^ at C1 (100% [95%CI 100–100] versus 83% [95%CI 67–99], log-rank *P* = 0.048) (Fig. 1D). Reaching MRD < 10^−4^ at C1 was not associated with pre-treatment biomarkers of high-risk disease (Supplementary Table 3).

We estimated the rate of disease eradication by calculating the log_10_ fold change in disease burden between baseline and C1 (ΔMRD). Mean ΔMRD was 5.0 log_10_ (i.e., >100.000-fold reduction), ranging from 2.6 to 7.3. Patients reaching MRD < 10^−5^ at EOiT had higher ΔMRD, compared to patients that failed to reach MRD < 10^−5^ (mean ΔMRD 5.1 versus 2.9, *P* = 0.02). The optimal ΔMRD cut-off to predict reaching MRD < 10^−5^ at EOiT was estimated at 4.0 log_10_ (*AUC* 0.93). ΔMRD was not associated with pre-treatment biomarkers of high-risk disease (Supplementary Table 4).

Besides MRD detection, IGHV sequencing captures the entire IGHV repertoire, a proxy of the diversity of the healthy B cell pool. As such, we investigated how treatment with obinutuzumab and venetoclax treatment affected the polyclonal IGHV background over time. IGHV repertoire diversity was analyzed in a total of 112 samples from 53/60 patients. After pre-induction with two cycles of obinutuzumab, patients at C1 had very low IGHV diversity, compared to healthy aged donors (mean Shannon’s *H* 0.69 [95%CI 0.44–0.95] versus 6.33 [95%CI 6.17–6.50], *P* < 0.0001). Following obinutuzumab cessation at cycle 6 and treatment with 6 cycles of venetoclax monotherapy, mean IGHV diversity increased incrementally to 1.38 (95%CI 0.94–1.83) at EOiT, 1.84 (95%CI 1.31–2.37) at R+6 and 4.04 (95%CI 2.74–5.34) at R+12 (Fig. 2A). Notably, at R+12, patients who had received venetoclax consolidation treatment had significantly lower IGHV diversity, compared to patients who had not received consolidation treatment (mean Shannon’s *H* 1.97 [1.41–2.53] versus 5.54 [3.56–7.52], *P* = 0.002) (Fig. 2B).

**Fig. 2:**
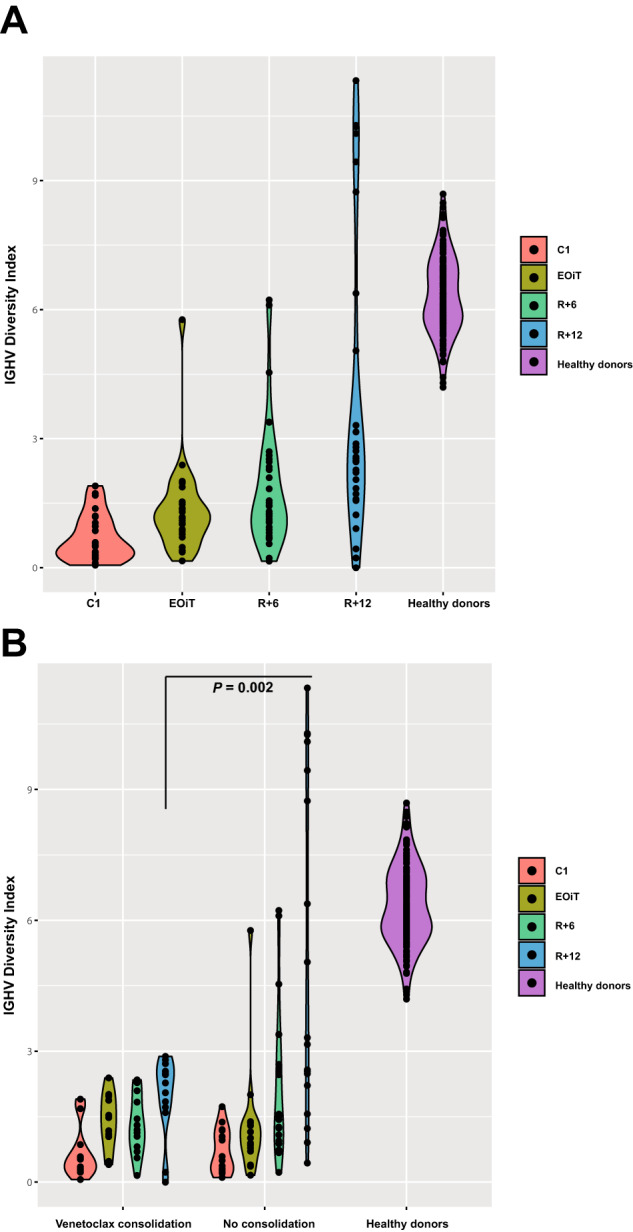
IGHV repertoire dynamics in the HOVON-139/GIVE trial. Dot- and violinplots depicting IGHV repertoire diversity over time of patients in the HOVON-139/GIVE trial (**A**), stratified by randomization arm in (**B**). Each dot represents one measurement. Violin width indicates density and can be interpreted as a mirrored histogram. IGHV diversity was quantified using Shannon’s diversity index. Statistical significance was evaluated using Welch’s *t*-test. C1 induction cycle 1, EOiT end of induction treatment, IGHV immunoglobulin heavy-chain variable, R+6 6 months after randomization, R+12 12 months after randomization.

In this study, we characterized the MRD dynamics and IGHV repertoire reconstitution of treatment-naive CLL patients treated with venetoclax and obinutuzumab in the HOVON-139/GIVE trial. Induction treatment induced deep and durable levels of MRD, resulting in uMRD (<10^−5^) in 93% of assessed patients, with 85 and 74% of patients retaining uMRD (<10^−5^) at 6 and 12 months after the end of induction therapy. Reaching low levels of MRD (<10^−4^) in the fourth week of induction cycle 1, having received 11 weeks of treatment, was associated with a 100% PFS rate in this cohort. Conversely, high MRD (>10^−2^) at C1 and a slow rate of early MRD eradication were associated with failure to achieve uMRD (<10^−5^) at the end of induction treatment.

Very early MRD measurements are a proxy for the rate of disease eradication. Our data indicate that a rapid reduction in disease burden can be used as a biomarker which, irrespective of established genomic features, signifies therapy-sensitive disease, resulting in deep MRD levels at the end of treatment and a long time to progression. Conversely, slow disease eradication is a prognostically poor sign. Whereas these patients still reach deep levels of MRD (<10^−4^ in this cohort), their response is shallower, culminating in the earlier loss of uMRD and a shorter time to progression. This notion is in line with the recent results of the UK TAP Clarity trial, where, in the relapsed and refractory setting, achieving a 100-fold or greater reduction of disease burden after 2 months of treatment with venetoclax and ibrutinib was associated with sustained low MRD and clinical response after a follow-up of 3 years [7, 8]. Moreover, in treatment-naïve patients receiving zanubrutinib, obinutuzumab and venetoclax, achieving a 2.6 log-reduction (400-fold) in MRD between baseline and cycle 5 was predictive of reaching undetectable MRD at cycle 8 and retaining MRD < 10^−5^ after 1 year of treatment cessation [9].

IGHV repertoire sequencing through the IGHV-leader NGS assay allows for simultaneous detection of MRD and characterization of the healthy polyclonal IGHV repertoire. High IGHV diversity is indicative of a large pool of non-malignant healthy B cells. As anticipated, following treatment with obinutuzumab and venetoclax, IGHV repertoire diversity was low. After cessation of obinutuzumab, IGHV repertoire diversity increased to near-normal levels in patients who did not receive venetoclax consolidation treatment. However, in patients who received venetoclax consolidation, reconstitution of the IGHV repertoire was impaired. Venetoclax is toxic to both CLL cells and healthy B cells [10]. Here, we demonstrate that consolidation treatment with venetoclax hampers recovery from the state of B cell aplasia induced by obinutuzumab, possibly resulting in a longer period of impaired humoral immunity. Whether this may have contributed to the higher rate of grade 2 or higher infectious adverse events in patients randomized to the venetoclax consolidation arm compared to the MRD-conditional arm (16/32 [50%] versus 6/30 [20%], as previously reported [5]), warrants a more in-depth analysis, incorporating more detailed clinical and serological data.

In conclusion, in treatment-naïve CLL patients treated with venetoclax and obinutuzumab, high levels of early MRD (>10^−2^) and a slow rate of MRD eradication, measured after 11 weeks of treatment, predicted failure to reach uMRD (<10^−5^) at the end of induction treatment. Conversely, patients with low levels of early MRD (<10^−4^) had a low probability of uMRD loss and disease progression. In addition, venetoclax consolidation treatment impaired polyclonal IGHV repertoire reconstitution after induction treatment with venetoclax and obinutuzumab, potentially associated with a higher chance of adverse infectious events.

## Methods

For the methods, please refer to the Supplementary Data.

## Supplementary information

Supplementary Data

Supplementary Figure 1

Supplementary Figure 2

Supplementary Figure 3

Supplementary Figure 4
