# Supplementary material for: Early-stage measurable residual disease dynamics and IGHV repertoire reconstitution during venetoclax and obinutuzumab treatment in chronic lymphocytic leukemia
Source: Blood Cancer J. 2023 Jul 4;13(1):102. doi: 10.1038/s41408-023-00870-2 (PMC10317999; doi:10.1038/s41408-023-00870-2)
Supplement: Supplementary file 1 — Supplementary Data [file 41408_2023_870_MOESM1_ESM.docx]

**Supplementary Methods**

*Patient samples and timepoints*

All patient material used in this study was obtained from the Dutch HOVON-139/GIVE trial (NTR6043), operated by the HOVON consortium. The HOVON-139/GIVE trial is a prospective, open-label, multicenter randomized phase-II trial intended to evaluate the efficacy and safety of pre-induction with two cycles of obinutuzumab, followed by induction with six cycles of obinutuzumab and venetoclax and six cycles of venetoclax alone, followed by either 12 cycles of venetoclax maintenance or MRD-conditional treatment cessation(1). Inclusion was restricted to previously untreated CLL patients, unfit for treatment with fludarabine, cyclophosphamide and rituximab-like regimens. More detailed information on in- and exclusion criteria and the trial protocol is available in the primary publication(1). MRD was measured by NGS at four timepoints: during the fourth week of venetoclax ramp up, at the end of induction treatment (after cycle 12), six months after randomization, and one year after randomization (i.e., at the end of optional consolidation treatment).

*IGHV-leader NGS*

MRD was quantified using the previously described IGHV-leader NGS assay(2). Briefly, IGHV libraries were amplified from genomic DNA, using the IGHV leader primer set developed by the EuroClonality-NGS working group(3). Per sample, we aimed for 6μg of DNA input, in PCR triplicates of 2 μg. After PCR cycling, libraries were checked using the D1000 DNA ScreenTape assay on a 4200 Agilent TapeStation (Agilent, Santa Clara, CA, USA) and purified using the AMPure XP kit (Beckman Coulter, Brae, CA, USA) with a volume ratio of 1.8X. PCR products were diluted to 4nM and equimolarly pooled in a single library-pool. Multiplexed libraries were denatured and sequenced at a final loading concentration of 8-20pM, with 5-20% of PhiX (Illumina, San Diego, CA, USA). Paired-end sequencing (2x300 cycles) was performed on a MiSeq system using a 600-cycle v3 kit (Illumina). NGS output was uploaded to the ARResT/Interrogate immunoprofiler(4). Reads were converted to cell-equivalents using the EuroClonality central in-tube quality control(5). The clonotypic IGH sequence, identified in a pre-treatment sample, was used to define malignant cell-equivalents. MRD depth was calculated by dividing the number of malignant cell-equivalents by the total number of cells equivalent to DNA input.

*Limit of detection and quantification*

As previously determined, the limit of detection and quantification (LoD/LoQ) of the IGHV-leader NGS assay are 3.4 and 3.8 malignant cell-equivalents per assay, respectively. Consequently, 6μg DNA input allows detection and quantification down to MRD 4,1*10^-6^. However, in some samples, final PCR input was either higher or lower (range 2-12μg, see **Supplementary Table 2**). To allow for uniform analysis of all samples, a LoD/LoQ cut-off was established at MRD 10^-5^. All samples assessable to at least MRD 10^-5^, where MRD levels were not detectable or <10^-5^, were designated as undetectable MRD (uMRD). Samples without detectable MRD that were not assessable to MRD 10^-5^ were excluded from the analysis.

*ΔMRD calculation*

The number of peripherally circulating leukemic cells at baseline was estimated by multiplying the total white blood cell count by the tumor burden percentage, measured by flow cytometry. The number of peripherally circulating leukemic cells at the fourth week of induction cycle 1 was estimated by multiplying the total white blood cell count, excluding neutrophils, by the MRD fraction measured by NGS. ΔMRD was defined as the log_10_-transformed fold change between these estimates. For samples with MRD at C1 below the LOD, ΔMRD was calculated by substituting the MRD fraction by the LOD (i.e., 10^-5^). The optimal ΔMRD cut-off was estimated using Youden’s index.

*IGHV repertoire diversity*

The workflow of the IGHV-leader NGS assay involves PCR-based amplification and sequencing of all IGHV rearrangements present in a sample. Not only does this capture information on the presence or absence of residual leukemic cells, but it also allows characterization of the healthy background IGHV repertoire. The sequence diversity of the IGHV repertoire, reflective of the size of the polyclonal healthy B cell pool, can be quantified using Shannon’s diversity index (*H*), which has 0 as minimal value (i.e., only one specie exists in the population), but has no theoretical upper limit (6). IGHV repertoire analysis was restricted to samples with at least 6μg DNA input. Samples from sequencing runs with an above-average error-rate were excluded from the analysis. Annotated IGHV repertoires were downloaded from ARResT/Interrogate and loaded to *R*, version 4.1.0(7). Shannon’s diversity index was calculated using the vegan package(8). Healthy donor IGHV repertoires had previously been obtained from a cohort of older individuals without signs or symptoms of any lymphoproliferative disease(9).

*Multicolor Flow-cytometry*

MRD measurements had been previously performed by multicolor flow cytometry (MFC) at the end of induction treatment, six months after randomization and one year after randomization, with a LoD of MRD 10^-4^ (10,11). In this approach, MRD is expressed as the number of residual leukemic cells as a fraction of the total leukocyte population, including granulocytes. To facilitate comparison to NGS measurements, MFC measurements were corrected using the concurrently measured granulocyte percentage.

*Statistical analysis*

Data analysis was performed in *R*. For continuous data, statistical significance was assessed using an unpaired Welch’s *t*-test. For categorical data, statistical significance was assessed using a χ^2^ test, or using Fisher’s exact test if these were fewer than 5 observations per condition. Pearson’s *r* was calculated using log_10_-transformed data. Kaplan-Meier survival analysis was performed using the survival package(12). Differences in survival time were evaluated using a log-rank test. Due to insufficient power, the trial protocol precludes any statistical comparison between the two randomization arms of the trial, regarding treatment efficacy and prognosis.

*Data availability statement*

IGHV sequencing data has been deposited in NCBI’s Gene Expression Omnibus and are accessible through GEO series accession number GSE225043(13). All other original data can be obtained upon reasonable request.

**Cross-validation with MFC**

We compared NGS and MFC measurements at all timepoints with MFC measurements available (146/185) (**Supplementary Figure 3**). MRD was detectable and quantifiable by both techniques in 9/146 (6%) samples (Pearson’s *r*=0.78, 95%CI [0.28-0.95], *P*=0.01). In 119/146 (82%) samples, MRD was undetectable by both techniques. ln 15/146 (10%) measurements, MRD was detectable by NGS, but not by MFC (MRD-NGS range: 2,6*10^-5^-1,07*10^-4^). In 3/146 (2%) measurements, MRD was detectable by MFC, but not by NGS (MFC-NGS range: 1,3*10^-4^-6,2*10^-4^). When reassessed by MFC at a later timepoint, MRD was no longer detectable (<10^-4^) in blood and bone marrow in any of these 3 patients. In between these measurements, 1/3 patients had received venetoclax consolidation, whereas 2/3 had not received any anti-leukemic treatment. The analyzed flow-cytometry plots of these three discordant samples are provided in **Supplementary Figure 4**.

**Supplementary Tables**

**Supplementary table 1.**

Baseline characteristics of the cohort of patients with available samples

|  | **Total cohort (n = 60)** |
| --- | --- |
| Sex (n, %)  *Male*  *Female* | 44 (73%)  16 (27%) |
| Age (years)  *Median (IQR)* | 71 (68-75) |
| RAI stage (n, %)  *0*  *I*  *II*  *III*  *IV* | 1 (2%)  12 (20%)  14 (23%)  16 (27%)  17 (28%) |
| CLL IPI score (n, %)  *1*  *2*  *3*  *4*  *NA* | 2 (3%)  10 (17%)  35 (58%)  9 (15%)  4 (7%) |
| IGHV mutational status (n, %)  *Unmutated*  *Mutated*  *NA* | 31 (52%)  24 (40%)  5 (8%) |
| Cytogenetic aberrations (n, %)  *del(13q14)*  *del(11q22)*  *del(17p13)*  *trisomy 12* | 40 (67%)  14 (23%)  9 (15%)  8 (13%) |
| TP53 mutations (n, %)  *Present*  *Absent* | 9 (15%)  51 (85%) |

**Supplementary table 2.**

Overview of DNA inputs used in this study.

| **DNA Input (μg)** | **Number of samples** |
| --- | --- |
| 2 - 4 | 5 |
| 4 – 5 | 10 |
| 5 | 29 |
| 6 | 144 |
| 10-12 | 10 |

**Supplementary table 3.**

Presence of pre-treatment biomarkers in patients that did or did not reach MRD <10^-4^ during induction cycle 1.

|  | **MRD ≥10^-4^ (n=22)** | **MRD <10^-4^ (n=12)** | ***P* value*** |
| --- | --- | --- | --- |
| IGHV mutational status  *Unmutated*  *Mutated* | 15  6 | 5  7 | 0.14 |
| Cytogenetic aberrations  *Del13q14*  *Del11q22*  *Del17p*  *Trisomy 12* | 14/22  4/22  5/22  2/22 | 6/12  1/12  2/12  4/12 | 0.5  0.6  0.99  0.15 |
| Genomic complexity  *≥3 aberrations* | 3/22 | 5/12 | 0.10 |
| TP53 gene mutation  *present* | 5/22 | 2/12 | 0.99 |

* Computed using Fisher’s exact test

**Supplementary table 4.**

Log_10_ fold change in disease burden between baseline and induction cycle 1, stratified by the presence of pre-treatment biomarkers.

|  | **Mean ΔMRD** | **95%CI of difference** | ***P* value*** |
| --- | --- | --- | --- |
| IGHV mutational status  *Unmutated*  *Mutated* | 5.0  5.3 | -0.7 – 1.4 | 0.5 |
| Del17p  *Present*  *Absent* | 5.0  5.1 | -1.4 – 1.3 | 0.9 |
| Genomic complexity  *≥3 aberrations*  *<3 aberrations* | 5.6  4.9 | -2.1 – 0.7 | 0.3 |
| TP53 gene mutation  *Present*  *Absent* | 5.0  5.1 | -1.4 – 1.3 | 0.9 |

* Computed using Welch’s two sample *t*-test

**Supplementary Figure Legends**

**Supplementary Figure 1. Schematic representation and overview of the samples used in this study**

Abbreviations: EOiT; end of induction treatment, MRD; measurable residual disease, R; randomization, R+6; six months after randomization, R+12; twelve months after randomization.

**Supplementary Figure 2. MRD dynamics per randomization arm**

Alluvial plots representing MRD dynamics of patients that received venetoclax consolidation treatment (A) and patients that did not receive venetoclax consolidation treatment (B). Counts indicate absolute patient numbers; ribbon sizes represent proportions.

Abbreviations: C1; induction cycle 1, EOiT; end of induction treatment, R + 6; six months after randomization, R + 12; twelve months after randomization

**Supplementary Figure 3. Comparison between IGHV leader-based NGS and MFC**

Comparison between MRD measurements obtained on samples from the HOVON-139/GIVE trial by multicolor flow cytometry and IGHV leader-based NGS. Each red dot represents one measurement. The horizontal and vertical black lines indicate the limits of detection of the NGS assay and MFC, respectively. Dots to the left of the vertical black lines signify uMRD by MFC, dots below the horizontal dotted line signify uMRD by IGHV leader-based NGS. The Pearson’s correlated coefficient was calculated using the log_10_-transformed values of samples in which MRD was quantifiable by both techniques. The black dotted line represents a perfect linear relationship.

Abbreviations: MFC; multicolor flow cytometry, MRD; measurable residual disease, uMRD; undetectable measurable residual disease, MRD+; detectable measurable residual disease, NGS; next-generation sequencing.

**Supplementary Figure 4. Analyzed flow-cytometry plots of the three samples with discordant NGS and MFC results**

**References**

1. Kersting S, Dubois J, Nasserinejad K, Dobber JA, Mellink C, van der Kevie-Kersemaekers A-MF, et al. Venetoclax consolidation after fixed-duration venetoclax plus obinutuzumab for previously untreated chronic lymphocytic leukaemia (HOVON 139/GiVe): primary endpoint analysis of a multicentre, open-label, randomised, parallel-group, phase 2 trial. Lancet Haematol. 2022;9(3):e190–9.

2. Hengeveld PJ, van der Klift MY, Kolijn PM, Davi F, Kavelaars FG, de Jonge E, et al. Detecting measurable residual disease beyond 10-4 through an IGHV leader-based NGS approach improves prognostic stratification in CLL. Blood. 2023;141(5):519–28.

3. Langlois de Septenville A, Boudjoghra M, Bravetti C, Armand M, Salson M, Giraud M, et al. Immunoglobulin gene mutational status assessment by next generation sequencing in chronic lymphocytic leukemia. In: Meth in Mol Biol, volume Immunogenetics. 2022. p. 2453:153-167.

4. Bystry V, Reigl T, Krejci A, Demko M, Hanakova B, Grioni A, et al. ARResT/Interrogate: an interactive immunoprofiler for IG/TR NGS data. Bioinformatics. 2017;33(3):435–7.

5. Knecht H, Reigl T, Kotrová M, Appelt F, Stewart P, Bystry V, et al. Quality control and quantification in IG/TR next-generation sequencing marker identification: protocols and bioinformatic functionalities by EuroClonality-NGS. Leukemia. 2019;

6. Shannon CE. A Mathematical Theory of Communication. Bell Syst Tech J. 27:623–56.

7. R Core Team. R: A language and environment for statistical computing. R Foundation for Statistical Computing [Internet]. 2020. Available from: https://www.r-project.org/

8. Oksanen J, Simpson GL, Blanchet FG, Kindt R, Legendre P, Minchin PR, et al. vegan. R package version 2.6-4 [Internet]. Available from: https://cran.r-project.org/web/packages/vegan/vegan.pdf

9. Kolijn PM, Saberi Hosnijeh F, Späth F, Hengeveld PJ, Andreas Agathangelidis, Saleh M, et al. High-risk subtypes of chronic lymphocytic leukemia are detectable as early as 16 years prior to diagnosis. Blood. 2021;

10. Rawstron AC, Villamor N, Ritgen M, Böttcher S, Ghia P, Zehnder JL, et al. International standardized approach for flow cytometric residual disease monitoring in chronic lymphocytic leukaemia. Leukemia. 2007;21(5):956–64.

11. Rawstron AC, Fazi C, Agathangelidis A, Villamor N, Letestu R, Nomdedeu J, et al. A complementary role of multiparameter flow cytometry and high-throughput sequencing for minimal residual disease detection in chronic lymphocytic leukemia: An European Research Initiative on CLL study. Leukemia. 2016;30(4):929–36.

12. Therneau T. A Package for Survival Analysis in R. R package version 3.4-0. [Internet]. 2022. Available from: https://cran.r-project.org/package=survival

13. Edgar R, Domrachev M, Lash AE. Gene Expression Omnibus: NCBI gene expression and hybridization array data repository. Nucleic Acids Res [Internet]. 2002 [cited 2021 Nov 11];30(1):207–10. Available from: http://www.ninds.nih.gov/
