# Supplementary material for: Early-stage measurable residual disease dynamics and IGHV repertoire reconstitution during venetoclax and obinutuzumab treatment in chronic lymphocytic leukemia
Source: Blood Cancer J. 2023 Jul 4;13(1):102. doi: 10.1038/s41408-023-00870-2 (PMC10317999; doi:10.1038/s41408-023-00870-2)
Supplement: Supplementary file 5 — Supplementary Figure 4 [file 41408_2023_870_MOESM5_ESM.pdf]

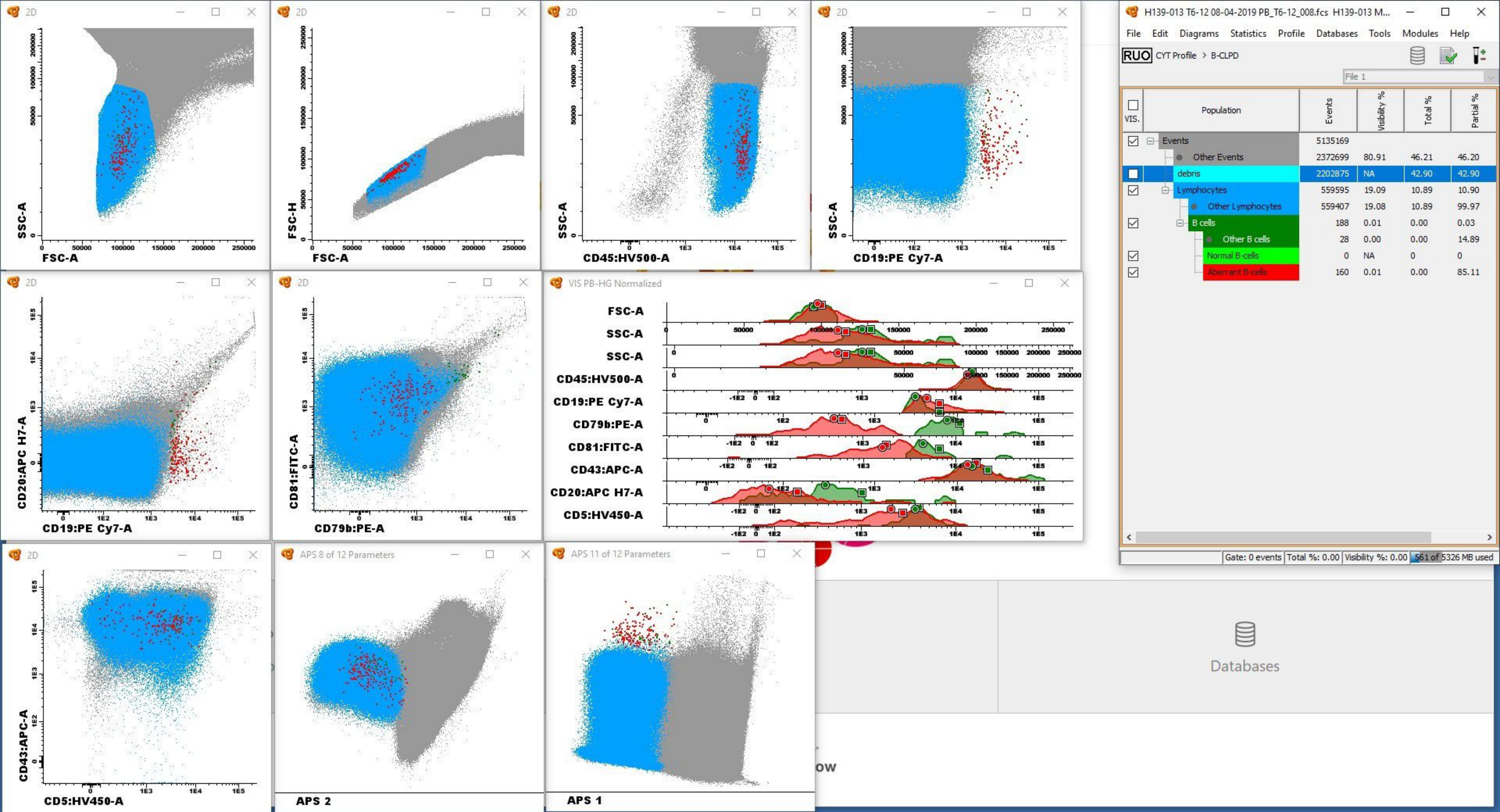
H139-013 T6-12 08-04-2019 PB_T6-12_008.fcs H139-013 M...
File Edit Diagrams Statistics Profile Databases Tools Modules Help
RUO CYT Profile > B-CLPD
File 1
VIS. Population Events Visibility % Total % Partial %
Events 5135169
Other Events 2372699 80.91 46.21 46.20
debris 2202875 NA 42.90 42.90
Lymphocytes 559595 19.09 10.89 10.90
Other Lymphocytes 559407 19.08 10.89 99.97
B cells 188 0.01 0.00 0.03
Other B cells 28 0.00 0.00 14.89
Normal B-cells 0 NA 0 0
Aberrant B-cells 160 0.01 0.00 85.11
Gate: 0 events Total %: 0.00 Visibility %: 0.00 561 of 5326 MB used
2D
FSC-A
SSC-A
FSC-H
CD45:HV500-A
CD19:PE Cy7-A
CD20:APC H7-A
CD79b:PE-A
CD81:FITC-A
VIS PB-HG Normalized
CD43:APC-A
CD5:HV450-A
APS 8 of 12 Parameters
APS 11 of 12 Parameters
APS 2
APS 1
Databases

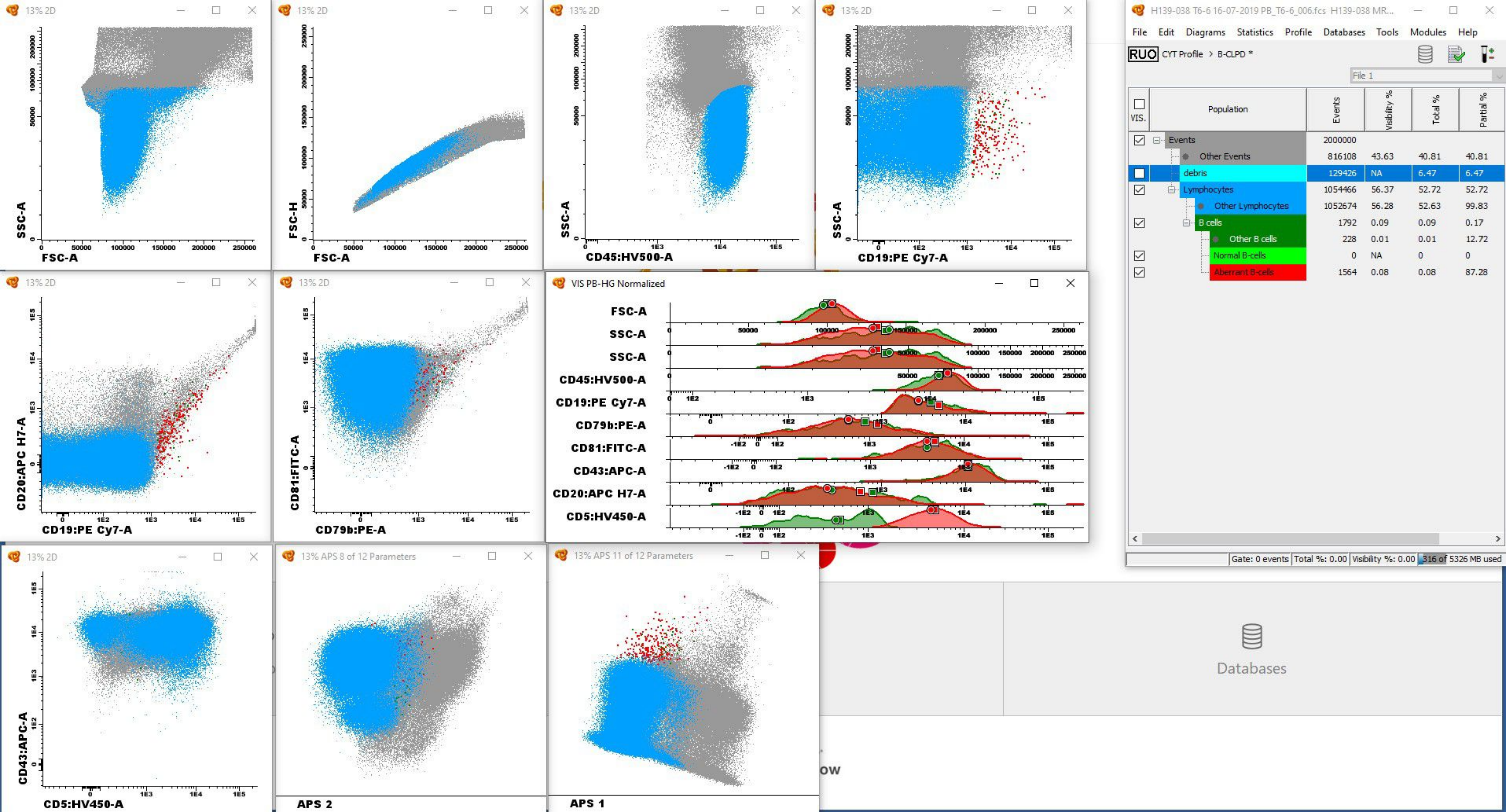
13% 2D
SSC-A
FSC-A
FSC-H
CD45:HV500-A
CD19:PE Cy7-A
CD20:APC H7-A
CD81:FITC-A
CD79b:PE-A
CD43:APC-A
CD5:HV450-A
VIS PB-HG Normalized
FSC-A
SSC-A
SSC-A
CD45:HV500-A
CD19:PE Cy7-A
CD79b:PE-A
CD81:FITC-A
CD43:APC-A
CD20:APC H7-A
CD5:HV450-A
13% APS 8 of 12 Parameters
APS 2
13% APS 11 of 12 Parameters
APS 1
H139-038 T6-6 16-07-2019 PB_T6-6_006.fcs H139-038 MR...
File Edit Diagrams Statistics Profile Databases Tools Modules Help
RUO CYT Profile > B-CLPD *
File 1
VIS. | Population | Events | Visibility % | Total % | Partial %
Events | 2000000
Other Events | 816108 | 43.63 | 40.81 | 40.81
debris | 129426 | NA | 6.47 | 6.47
Lymphocytes | 1054466 | 56.37 | 52.72 | 52.72
Other Lymphocytes | 1052674 | 56.28 | 52.63 | 99.83
B cells | 1792 | 0.09 | 0.09 | 0.17
Other B cells | 228 | 0.01 | 0.01 | 12.72
Normal B-cells | 0 | NA | 0 | 0
Aberrant B-cells | 1564 | 0.08 | 0.08 | 87.28
Gate: 0 events | Total %: 0.00 | Visibility %: 0.00 | 316 of 5326 MB used
Databases

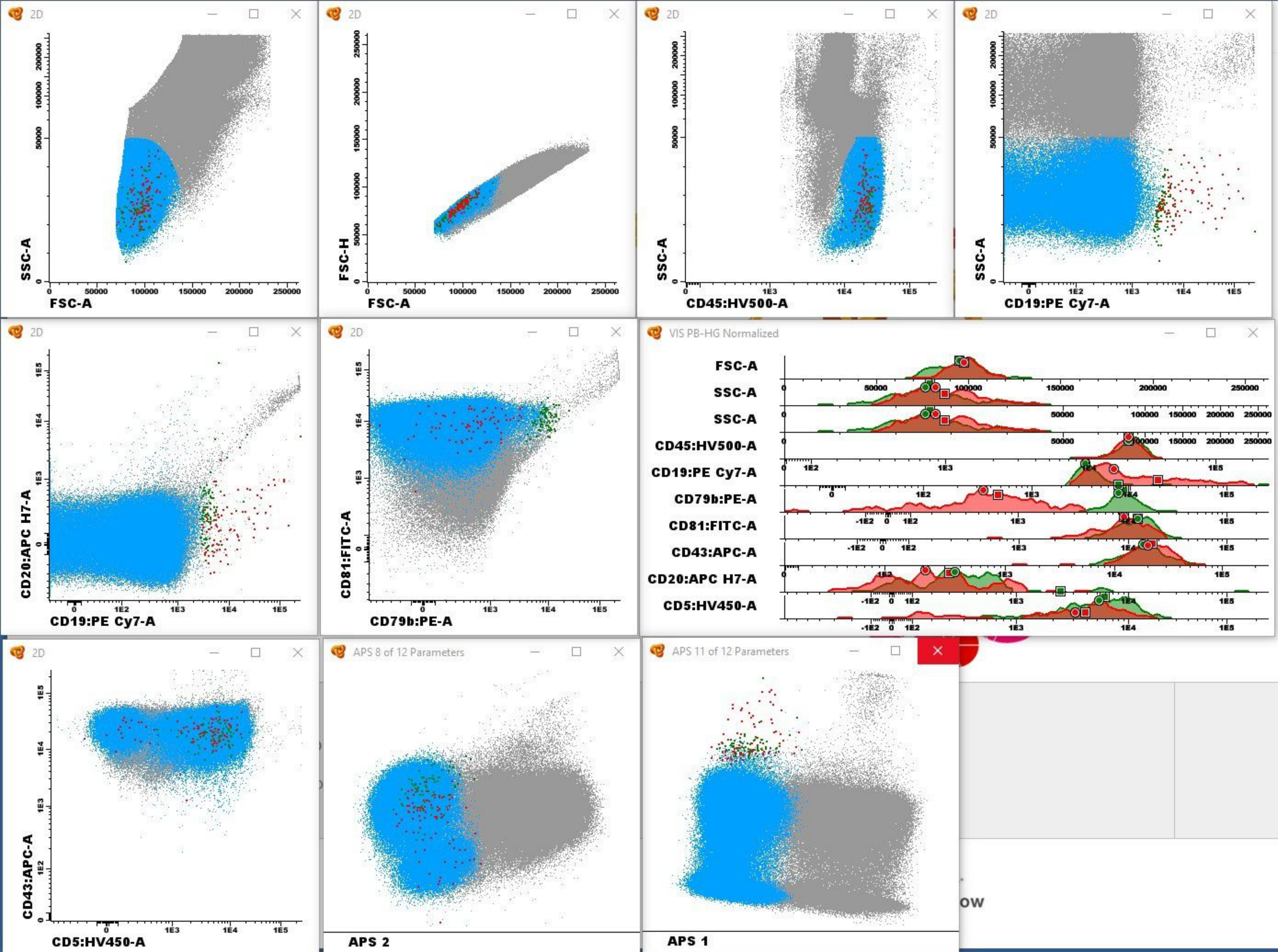

H139-010 T5 19-04-2018 PB_T5 PB_003.fcs H139-010 MRD...

File Edit Diagrams Statistics Profile Databases Tools Modules Help

RUO CYT Profile > B-CLPD

File 1

VIS.	Population	Events	Visibility %	Total %	Partial %
☑	Events	427004			
	Other Events	193598	49.91	45.34	45.34
☐	debris	39127	NA	9.16	9.16
☑	Lymphocytes	194279	50.09	45.50	45.50
	Other Lymphocytes	194117	50.05	45.46	99.92
☑	B cells	162	0.04	0.04	0.08
	Other B cells	79	0.02	0.02	48.77
☑	Normal B-cells	0	NA	0	0
☑	Aberrant B-cells	83	0.02	0.02	51.23

Gate: 0 events | Total %: 0.00 | Visibility %: 0.00 | 161 of 5326 MB used

Databases

ow
