# Supplementary material for: Early-stage measurable residual disease dynamics and IGHV repertoire reconstitution during venetoclax and obinutuzumab treatment in chronic lymphocytic leukemia
Source: Blood Cancer J. 2023 Jul 4;13(1):102. doi: 10.1038/s41408-023-00870-2 (PMC10317999; doi:10.1038/s41408-023-00870-2)

Supplementary Figure 3

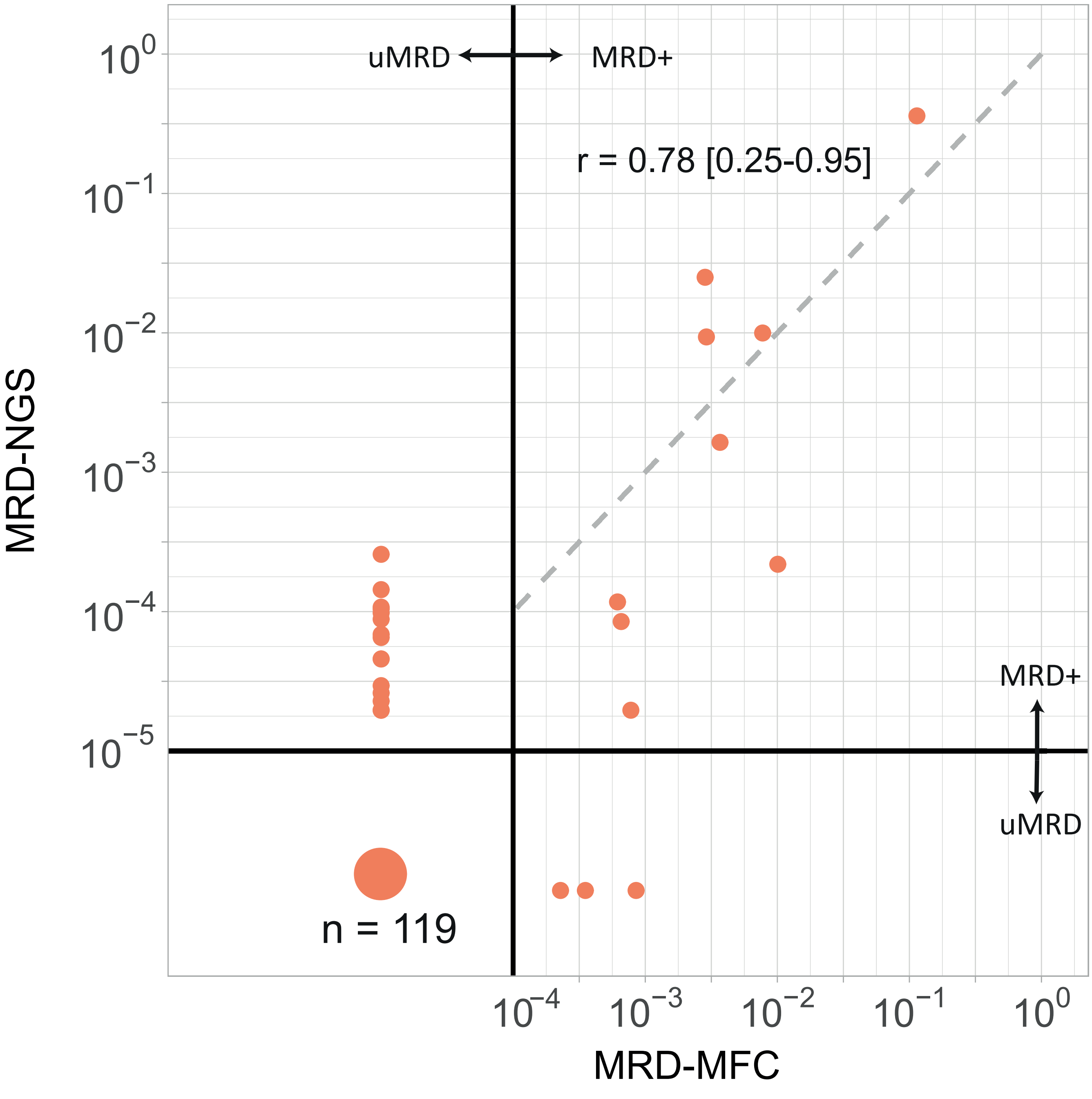

Supplement: Supplementary file 4 — Supplementary Figure 3 [file 41408_2023_870_MOESM4_ESM.pdf]
